# Molecular diagnosis and phylogenetic analysis of *Babesia bigemina* and *Babesia bovis* hemoparasites from cattle in South Africa

**DOI:** 10.1186/1746-6148-9-154

**Published:** 2013-08-08

**Authors:** Moses Sibusiso Mtshali, Phillip Senzo Mtshali

**Affiliations:** 1 Research and Scientific Services Department, Veterinary Parasitology Unit, National Zoological Gardens of South Africa, Pretoria 0001, South Africa; 2 Department of Zoology and Entomology, Parasitology Research Program, University of the Free State, Qwaqwa Campus, Phuthaditjhaba 9866, South Africa

**Keywords:** Cattle, *Babesia bigemina*, *Babesia bovis*, South Africa, Nested PCR, Phylogeny

## Abstract

**Background:**

*Babesia* parasites, mainly *Babesia bovis* and *B. bigemina*, are tick-borne hemoparasites inducing bovine babesiosis in cattle globally. The clinical signs of the disease include, among others, anemia, fever and hemoglobinuria. Babesiosis is known to occur in tropical and subtropical regions of the world. In this study, we aim to provide information about the occurrence and phylogenetic relationship of *B. bigemina* and *B. bovis* species in cattle from different locations in nine provinces of South Africa.

A total of 430 blood samples were randomly collected from apparently healthy cattle. These samples were genetically tested for Babesia parasitic infections using nested PCR assays with species-specific primers.

**Results:**

Nested PCR assays with Group I primer sets revealed that the overall prevalence of *B. bigemina* and *B. bovis* in all bovine samples tested was 64.7% (95% CI = 60.0-69.0) and 35.1% (95% CI = 30.6-39.8), respectively. Only 117/430 (27.2%) animals had a mixed infection. The highest prevalence of 87.5% (95% CI = 77.2-93.5) for *B. bigemina* was recorded in the Free State province collection sites (Ficksburg, Philippolis and Botshabelo), while North West collection sites had the highest number of animals infected with *B. bovis* (65.5%; 95% CI = 52.7-76.4). Phylograms were inferred based on *B. bigemina*-specific *gp45* and *B. bovis*-specific *rap-1* nucleotide sequences obtained with Group II nested PCR primers. Phylogenetic analysis of *gp45* sequences revealed significant differences in the genotypes of *B. bigemina* isolates investigated, including those of strains published in GenBank. On the other hand, a phylogeny based on *B. bovis rap-1* sequences indicated a similar trend of clustering among the sequences of *B. bovis* isolates investigated in this study.

**Conclusion:**

This study demonstrates the occurrence of *Babesia* parasites in cattle from different provinces of South Africa. It was also noted that the situation of *Babesia* parasitic infection in cattle from certain areas within the surveyed provinces had either reached endemic stability or was progressing towards stability.

## Background

Bovine babesiosis (also known as redwater) is one of the most economically important infectious diseases affecting cattle worldwide. This tick-borne disease is mainly induced by intraerythrocytic protozoan parasites of the genus *Babesia*, order Piroplasmida and phylum Apicomplexa [1]. In southern Africa, *Babesia bigemina* and *Babesia bovis* are the two economically important species infecting cattle and have high prevalence in tropical and subtropical regions of the world [2]. Clinical signs characterizing the disease caused by the two parasites are anemia, fever, hemoglobinuria, and in many cases death [3,4].

Traditionally, the microscopic detection of *Babesia* parasites has always been considered as the gold standard for the diagnosis of acute babesiosis [5]. However, the major drawback associated with microscopic examination of blood parasites is the low sensitivity offered by the technique, thus making it difficult to detect parasites in blood smears during low parasitemia in the case of carrier animals [6]. Alternatively, PCR-based assays have been widely used for the detection of *Babesia* parasites owing to their high specificity and sensitivity [4,6-9].

At present, there are still limited tools available for the prevention and eradication of bovine babesiosis. By far, the most recommended approach for controlling ticks and tick-borne diseases in South Africa is by integrating the strategic use of acaricides (i.e. the pesticides used for killing ticks) and application of vaccines [10]. However, more than 90% of resource-poor farmers contend that the dip wash is not effective in killing the ticks. Consequently, these farmers complement the government dipping service with their own initiatives, which include spraying with conventional acaricides, using household disinfectants and manual removal [11,12].

It is estimated that, in South Africa, approximately 18% of all cattle mortalities are due to tick-borne diseases including babesiosis, anaplasmosis and heartwater [10]. These diseases have a considerable impact on the country’s economic security and also impact negatively on poor communities who are dependent on livestock production as their source of income and nutritional needs (meat and milk), and as labour for fieldwork and transport [13]. As such, it is reported that the occurrence of *B. bigemina* and *B. bovis* in South African cattle population hampers the development of livestock industry, which accounts for up to 49% of the agricultural output [14].

Given the importance of livestock production in the South African economic landscape, in this study, we genetically investigated the occurrence and distribution of *Babesia* parasites, more specifically *B. bigemina* and *B. bovis*, in bovine samples randomly collected from different locations throughout South Africa. We also studied the phylogenetic relatedness amongst DNA sequences of randomly selected bovine samples.

## Methods

### Blood sample collection

Experimental collection of blood samples from cattle was approved by the NZG Ethics and Scientific Committee, National Zoological Gardens of South Africa. Between 2010 and 2012, a total of 430 blood samples were randomly collected from clinically healthy cattle occupying different locations in all nine provinces of South Africa: Mpumalanga province (Ehlanzeni South District; *n* = 48), KwaZulu-Natal province (Albert Falls, Ndaleni dip tank and Shallow Drift; *n* = 52), Limpopo province (Capricorn District; *n* = 47), North West province (Maubane and Mmatlhwaela; *n* = 58), Gauteng province (Bronkhorstspruit; *n* = 30), Free State province (Ficksburg, Philippolis and Botshabelo; *n* = 64), Eastern Cape province (Alice, Fort Beaufort and Adelaide; *n* = 60), Northern Cape province (Kuruman; *n* = 45) and Western Cape province (Boland area; *n* = 26). The total number of animals sampled in each collection site depended on the number of cattle present at the sampling stations (dip tanks/cattle farms). No information on the age groups, husbandry practices, vaccination histories and tick infestation status of the sampled cattle were available, given that other bovine blood samples were collected and provided by the farmers. Blood was collected from the coccygeal vein into EDTA-coated vacutainer tubes, transported to the laboratory on ice and stored at −20°C until further analysis.

### DNA extraction

Genomic DNA was extracted from 200 μl of blood using ZR Genomic DNA™-Tissue MiniPrep kit (Inqaba Biotechnical Industries, Pretoria, South Africa) according to the manufacturer’s instructions. Extracted DNA was eluted in 50 μl of DNA elution buffer and stored at −20°C until further analysis. DNA concentration was determined using a NanoDrop® ND-1000 (NanoDrop Technologies Inc., Wilmington, USA).

### Primer design

Four sets of oligonucleotide primers reported previously [8] were employed for detecting *B. bigemina* and *B. bovis* parasites in field blood samples. To obtain DNA sequences for phylogenetic analysis, new sets of species-specific primers were designed (Table 1). These primers targeted merozoite surface glycoprotein 45 (*gp45*) and rhoptry-associated protein 1 (*rap-1*) genes specific for *B. bigemina* and *B. bovis*, respectively. The *gp45* gene sequences of *B. bigemina* used for primer design were extracted from GenBank under accession numbers JN049649–JN049655 and AF298631–AF298632. The GenBank accession numbers for *B. bovis rap-1* sequences were FJ588009–FJ588013 and AF030056–AF030060.

**Table 1:** Primary and nested PCR primers used for PCR amplifications

**Species**	**Assay**	**Primer sequence (5′ → 3′)**	**Annealing**	**Product size**
**Oligonucleotides used for screening (Group I)**^**a**^				
*B. bigemina*	PCR	**F**-CATCTAATTTCTCTCCATACCCCTCC	55°C	278 bp
		**R**-CCTCGGCTTCAACTCTGATGCCAAAG		
	nPCR	**F**-CGCAAGCCCAGCACGCCCCGGTGC	55°C	170 bp
		**R**-CCGACCTGGATAGGCTGTGTGATG		
*B. bovis*	PCR	**F**-CACGAGGAAGGAACTACCGATGTTGA	55°C	360 bp
		**R**-CCAAGGAGCTTCAACGTACGAGGTCA		
	nPCR	**F**-TCAACAAGGTACTCTATATGGCTACC	55°C	298 bp
		**R**-CTACCGAGCAGAACCTTCTTCACCAT		
**Oligonucleotides used for phylogenetic study (Group II)**^**b**^				
*B. bigemina*	PCR	**F**-GTGCTGCTTAATCGCACAAAC	55°C	963 bp
		**R**-AAGATGCCTTCTTCGGTGATG		
	nPCR	**F**-CGGATCCTGTTATCGTTCCTG	56°C	853 bp
		**R**-GAAGTTACGCCTGGAGTTGG		
*B. bovis*	PCR	**F**-TCAGATTGTTCAAAGAGAGTGCATCC	55°C	1280 bp
		**R**-GTCTTCACCGTTGGAAGTAGTTGAGTC		
	nPCR	**F**-CACGAGGAAGGAACTACCGATGTTGA	64°C	1009 bp
		**R**-CCTTTGTAGGTTGGCCAACAGTTTCG		

^a^ Group I oligonucleotide sequences were taken from published work [8].

^b^ Group II primers were designed in this study.

The new pairs of PCR and nested PCR primers were designed from conserved regions identified after performing multiple sequence alignments with CLUSTAL W algorithm [15] embedded in BioEdit software [16]. The specificity of newly designed primers was tested against GenBank sequences using BLAST search [17]. All primers were synthesized by Inqaba Biotechnical Industries.

### Specificity of nested PCR

Purified DNA samples of *B. bigemina*, *B. bovis*, *Anaplasma centrale*, *Theileria parva* and *Ehrlichia ruminantium* were used to assess the specificity of Group I and Group II primers. These DNA samples were kindly provided by Dr Nicola Collins (Department of Veterinary Tropical Diseases, University of Pretoria, South Africa) and Dr Oriel Thekisoe (Parasitology Research Program, University of the Free State, South Africa). PCR and nested PCR mixtures were prepared and thermally cycled as described above using 2 μl of each of the purified DNA samples.

### PCR detection assays

To detect the presence of *B. bigemina* and *B. bovis* from bovine samples using Group I primers, PCR was performed in a 25-μl reaction mixture containing 5 μl of the extracted DNA, 0.6 μM of each primer and 12.5 μl of DreamTaq Green PCR Master Mix (Inqaba Biotechnical Industries). Negative control reactions contained distilled water instead of template DNA. Reaction mixtures were subjected to PCR using BIO-RAD T100™ Thermal Cycler (Bio-Rad Laboratories, Johannesburg, South Africa). PCR amplifications (round 1) were performed at the following thermal conditions: 95°C for 3 min, followed by 35 cycles of 95°C for 30 sec, 55°C for 45 sec and 72°C for 1 min. Following the final extension step at 72°C for 10 min, 1 μl of each PCR product was added into the second (nested) PCR mixture comprising similar composition of reagents as the first round PCR, except that the external primers were replaced with the nested PCR primers. PCR mixtures were cycled as described above using annealing temperatures reflected in Table 1.

PCR-generated amplicons were analyzed by electrophoresis in 1.5% agarose gels stained with Biotium GelRed Acid Stain (Anatech Instruments, Johannesburg, South Africa) and visualized under UV illumination. GeneRuler™ 1 kb Plus DNA ladder (Inqaba Biotechnical Industries) was used as the standard molecular weight marker.

### Sequencing and phylogenetic analysis

For phylogenetic study, genomic DNA of randomly selected field samples was amplified with Group II nested PCR primers (Table 1) targeting *gp45* and *rap-1* fragments of *B. bigemina* and *B. bovis*, respectively. Seven bovine samples were selected for *B. bigemina* and six for *B. bovis*. PCR mixtures were prepared and thermally cycled as described above employing the annealing temperatures reflected in Table 1. PCR-generated fragments of 853 bp (for *B. bigemina*) and 1009 bp (for *B. bovis*) were sent to Inqaba Biotechnical Industries for purification and sequencing in both directions using ABI 3130 XL Genetic Analyzer (Applied Biosystems, Johannesburg, South Africa). At least two individually amplified DNA fragments of each selected sample were sequenced.

Nucleotide sequences were assembled and aligned using BioEdit software program [16]. The resulting consensus sequences were subsequently used to search for homologous sequences in GenBank. To construct phylogenetic trees, the consensus nucleotide sequences were trimmed manually to equivalent lengths. Phylogenies were inferred using the neighbour-joining algorithm of the MEGA v4.1 software [18]. Bootstrapping analysis with 1000 replicates was used to estimate the robustness of individual branches [19].

### Nucleotide sequence accession numbers

The determined *B. bigemina gp45* and *B. bovis rap-1* gene sequences were submitted to GenBank database under the accession numbers KC894392-KC894404.

### Statistical analysis

The proportions for 95% confidence intervals (95% CI) were computed as CIs for proportions with binomial data employing no continuity correction.

## Results and discussion

Blood samples collected from 430 cattle were genetically tested for the presence of *Babesia* parasites. Nested PCR assays with Group I primers developed previously [8] were employed to detect *B. bigemina* and *B. bovis* pathogens in bovine samples collected from different locations throughout South Africa. The samples presenting single amplification fragments of approximately 170 bp and 298 bp were considered positive for *B. bigemina* and *B. bovis*, respectively. The results of nested PCR amplification assays are presented in Table 2.

**Table 2:** **Nested PCR detection results of *B. bigemina *and *B. bovis *parasites in field blood samples**

**Province**	**Total number of samples**	***Babesia bigemina***		***Babesia bovis***		**Mixed infection**	
		**No. positive**	**Percentage**	**No. positive**	**Percentage**	**No. positive**	**Percentage**
Mpumalanga	48	32	66.7	21	43.8	15	31.3
KwaZulu-Natal	52	44	84.6	33	63.5	28	53.9
Limpopo	47	39	83.0	11	23.4	11	23.4
North West	58	41	70.7	38	65.5	24	41.4
Gauteng	30	25	83.3	19	63.3	18	60.0
Free State	64	56	87.5	15	23.4	14	21.9
Eastern Cape	60	21	35.0	7	11.7	4	6.7
Northern Cape	45	2	4.4	2	4.4	0	0
Western Cape	26	18	69.2	5	19.2	3	11.5
**Total**	**430**	**278**	**64.7**	**151**	**35.1**	**117**	**27.2**

The animals infected with *B. bigemina* and *B. bovis* were found in all the provinces surveyed, albeit there were differences observed in the distribution of these hemoparasites in cattle from certain provinces. The occurrence of *B. bigemina* and *B. bovis* in cattle from all surveyed areas could be attributed to the presence and distribution of tick vectors transmitting these parasites. Nested PCR assays detected mostly *B. bigemina* compared to *B. bovis*, and these results are similar to previous findings reported elsewhere [20-22]. However, a notable exception was evident for blood samples collected from Kuruman in the Northern Cape province where the occurrence of both *Babesia* species was below 5%. This low prevalence is not unusual for an area that is considered free of babesiosis tick vectors.

The overall prevalence of *B. bigemina* and *B. bovis* in all samples was 64.7% (95% CI = 60.0-69.0) and 35.1% (95% CI = 30.6-39.8), respectively. Samples from the Free State province had the highest frequency of *B. bigemina* (87.5%, 95% CI = 77.2-93.5), followed by KwaZulu-Natal province with as high as 84.6% (95% CI = 72.5-92.0) of samples tested positive for *B. bigemina*. These high prevalence values (>80%) were indicative of an endemically stable situation [23]. By definition, an endemically stable situation occurs when 81–100% of the herd are infected with a particular *Babesia* species [23]. The highest number of samples positive for *B. bovis* was recorded in the North West province collection sites (65.5%, 95% CI = 52.7-76.4), thus indicating the situation approaching endemic stability but with the potential occurrence of disease outbreaks [23]. Of all the collection sites surveyed, Kuruman area in Northern Cape province had the lowest prevalence of 4.4% (2 out of 45; 95% CI = 1.2-14.8) for both *B. bigemina* and *B. bovis*. The cattle found to contain DNA of both *B. bigemina* and *B. bovis* (117/430, 27.2%) originated from all but one of the nine provinces from which blood samples were collected. None of the blood samples collected from Kuruman (Northern Cape province) possessed mixed infections.

In principle, the occurrence of *B. bigemina* and *B. bovis* in cattle is largely dependent on the distribution of tick vectors [24]. The sole vector of *B. bovis* in South Africa is *Rhiphicephalus* (*Boophilus*) *microplus*, whereas *B. bigemina* is transmitted by *R.* (*B.*) *microplus*, *Rhiphicephalus* (*Boophilus*) *decoloratus* and *Rhiphicephalus evertsi evertsi*[3]. Based on the geographical distribution of tick species in South Africa, *R.* (*B.*) *microplus* is more prevalent in KwaZulu-Natal, Eastern Cape and Limpopo provinces, while the high prevalence of *R.* (*B.*) *decoloratus* was recorded in Mpumalanga, Eastern Cape and KwaZulu-Natal provinces. On the other hand, *R. evertsi evertsi* is reported to be present throughout South Africa, except in arid areas of the Northern Cape and northern Western Cape provinces [25-29].

Therefore, it is not surprising that in the present study, there was a high percentage of bovine samples positive for *B. bigemina* and *B. bovis* infections. These results confirm the previous findings that *Babesia* infections are common in cattle in South Africa [14,30,31]. Using serology-based assays, a recent study [14] demonstrated the existence of *B. bigemina* and *B. bovis* parasites in cattle from eight South African provinces surveyed. Surprisingly, more than 20% of animals from Northern Cape province were found to possess *B. bigemina* and *B. bovis* when tested with IFAT and ELISA [14], and these values are considered higher for an area known to be free of *R.* (*B.*) *microplus*, *R.* (*B.*) *decoloratus* and *R. evertsi evertsi* tick vectors. According to the latter authors [14], the higher prevalence values could be explained by the possible outsourcing of animals from endemic areas in the neighbouring provinces.

To study the phylogenetic relationship between *Babesia* parasites of randomly selected bovine samples, new sets of nested PCR primers (Group II) were designed based on nucleotide sequences of the *gp45* (*B. bigemina*-specific) and *rap-1* (*B. bovis*-specific) genes. The specificity of both Group I and Group II primers was tested against purified DNA samples of *B. bigemina*, *B. bovis*, *A. centrale*, *T. parva* and *E. ruminantium*. As expected, the nested PCR assay with *B. bigemina* gene-specific primers only detected the DNA of *B. bigemina*. Likewise, *B. bovis*-specific nested PCR assay specifically identified *B. bovis* DNA sample, and no amplifications were observed for DNA samples derived from *B. bigemina*, *A. marginale, T. parva* and *E. ruminantium*.

**Figure 1:**
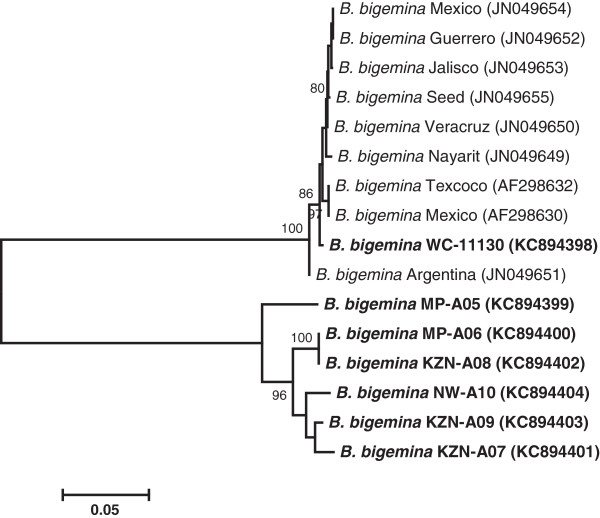
**Phylogenetic tree inferred from partial *gp45 *nucleotide gene sequences (818–827 *nt*) and showing the relationship between *B. bigemina *isolates investigated in this study (indicated in bold) and other *B. bigemina *strains published in GenBank (accession numbers in parentheses).** The tree was constructed using the neighbour-joining algorithm of MEGA v4.1 software. The numbers at the nodes are bootstrap values expressed as percentages of 1000 replicates; only the values above 80% are shown. The bar (0.05) represents the number of mutations per site. Isolate designations: MP - Mpumalanga province, NW - North West province, WC - Western Cape province, and KZN - KwaZulu-Natal province.

The neighbour-joining tree inferred with *gp45* gene sequences of *B. bigemina* isolates determined in this study is shown in Figure 1. The closely related sequences of *B. bigemina gp45* genes retrieved from GenBank were also incorporated in the phylogeny. From the phylogenetic analysis, it was worth noting that our isolates were clearly distinct from other closely related taxa whose sequences were obtained from GenBank database (Figure 1). Interestingly, WC-11130 isolate (accession no. KC894398) clustered with *B. bigemina* strains from GenBank, suggesting the high genetic similarity of our isolate with published strains. As highlighted in the literature, the diversity observed between *gp45* sequences of *B. bigemina* is not unusual. The high polymorphism of gp45 B-cell epitopes amongst the American isolates of *B. bigemina* was reported previously [32]. Given the nucleic acid sequence variations observed amongst the tested *B. bigemina* isolates, it may be expected that these sequence heterogeneities would induce changes in the protein structure.

**Figure 2:**
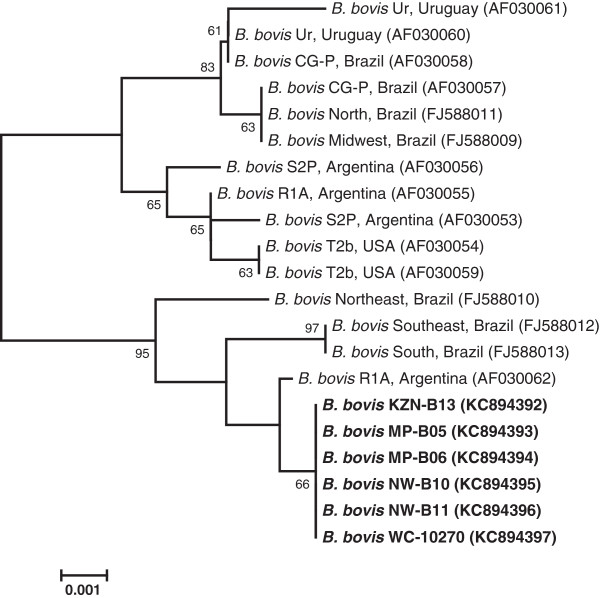
**Phylogenetic tree based on *rap-1 *gene sequences (947 *nt*) of *B. bovis *isolates identified in this study (indicated in bold) and those of strains whose sequences were extracted from GenBank (accession numbers in parentheses).** The tree was constructed using the neighbour-joining method, with bootstrap values (expressed as percentages of 1000 replications) superimposed at branching points; only the values above 60% are shown. The horizontal bar represents the number of base substitutions per site. Isolate designations: MP - Mpumalanga province, NW - North West province, WC - Western Cape province, and KZN - KwaZulu-Natal province.

In a phylogram generated with *rap-1* gene sequences of *B. bovis*, as presented in Figure 2, it appears that *B. bovis* isolates tested in this study are phylogenetically similar to *B. bovis* strains originating from countries other than South Africa. Results on the genetic conservation between *rap-1* sequences of *B. bovis* strains have also been reported elsewhere [33,34]. Nevertheless, our isolates formed a monophyletic grouping clearly distinct from that of other published *B. bovis* strains, and this suggested the presence of micro-heterogeneities among the *rap-1* sequences within *B. bovis* species.

## Conclusion

The results presented in this study demonstrate a high incidence of cattle infection by *B. bigemina* and *B. bovis* in sampling areas from all provinces surveyed, with a notable exception of Kuruman area (Northern Cape province) that displayed a very low occurrence of *Babesia* parasites. The high prevalences (>80%) of *B. bigemina* in cattle from KwaZulu-Natal, Limpopo, Gauteng and Free State sampling areas suggest that the situation of *B. bigemina* infection has reached endemic stability. Conversely, the prevalence of *B. bovis* in bovine samples from KwaZulu-Natal, North West and Gauteng collection sites suggests that the situation in these areas is progressing towards endemic stability. Therefore, in order to attain endemic stability to *Babesia* parasites, a limited number of tick vectors should be allowed to infest cattle, particularly in the case of herds occupying areas with lower infection rates.

In addition, this study has expanded our current knowledge concerning the genetic diversity and phylogenetic relatedness among *B. bigemina* and *B. bovis* isolates of South African origin. Given the limited number of *Babesia*-specific gene sequences available in GenBank, particularly those of *Babesia* species originating from South African cattle, further studies incorporating sampling sites representative of each surveyed province would be required. Undoubtedly, this will enable a better understanding of the epidemiology of bovine babesiosis as well as the degree of genetic heterogeneities among *B. bigemina* and *B. bovis* isolates in South African cattle. Overall, the findings from this study will ultimately help farmers develop efficient control strategies to curtail cattle mortalities emanating from bovine babesiosis.

## Competing interests

The authors declare that they have no competing interests.

## Authors’ contributions

MS conceived and designed the study. PS performed laboratory experiments, participated in data analysis, constructed and interpreted phylogenetic trees, and drafted the manuscript. MS and PS critically reviewed the first draft of the manuscript. All authors read and approved the final version of the manuscript.
